# Persistence of the 2009 Pandemic Influenza A (H1N1) Virus in Water and on Non-Porous Surface

**DOI:** 10.1371/journal.pone.0028043

**Published:** 2011-11-23

**Authors:** Amélie Dublineau, Christophe Batéjat, Anthony Pinon, Ana Maria Burguière, India Leclercq, Jean-Claude Manuguerra

**Affiliations:** 1 Institut Pasteur, Laboratory for Urgent Response to Biological Threats, Paris, France; 2 Institut Pasteur de Lille, Microbiological Safety Unit, Lille, France; 3 University Paris Diderot, Sorbonne Paris Cité (Cellule Pasteur), Paris, France; The University of Hong Kong, China

## Abstract

Knowledge of influenza A virus survival in different environmental conditions is a key element for the implementation of hygiene and personal protection measures by health authorities. As it is dependent on virus isolates even within the same subtype, we studied the survival of the 2009 H1N1 pandemic (H1N1pdm) virus in water and on non-porous surface. The H1N1pdm virus was subjected to various environmental parameters over time and tested for infectivity. In water, at low and medium salinity levels and 4°C, virus survived at least 200 days. Increasing temperature and salinity had a strong negative effect on the survival of the virus which remained infectious no more than 1 day at 35°C and 270 parts per thousand (ppt) of salt. Based on modeled data, the H1N1pdm virus retained its infectivity on smooth non-porous surface for at least 7 days at 35°C and up to 66 days at 4°C. The H1N1pdm virus has thus the ability to persist in water and on glass surface for extended periods of time, even at 35°C. Additional experiments suggest that external viral structures in direct contact with the environment are mostly involved in loss of virus infectivity.

## Introduction

The threat of pandemic Highly Pathogenic Avian Influenza Virus (HPAIV) H5N1 and the recent outbreak caused by the novel 2009 A(H1N1) influenza virus generated a renewed interest in the study of influenza virus transmission. Aerosols, large droplets and contact of the nasal mucosa by contaminated hands all contribute to the transmission of influenza viruses [1], [2]. Virus survival in different environmental settings is a key element for control measures and decisions introduced by national health authorities and policy makers. Due to the H5N1 outbreak threat, efforts have been made to understand the survival of influenza viruses in the environment. But, in spite of a recent and sharp increase of published papers on influenza virus survival, knowledge still remains scarce. The majority of studies were done in water with avian influenza viruses, which are transmitted through an indirect fecal-oral route involving fecal-contaminated waters [3], [4]. They showed that avian influenza viruses can remain infective for extended durations in water, from 100 days to several months depending on the temperature [5], [6]. Works focused on HPAIV H5N1 strengthened the evidence of increased stability in water of influenza viruses at cold temperatures, but were contradictory regarding the effect of high temperature on H5N1 survival [5], [7]. Furthermore, less information is available regarding the survival of influenza viruses in the air and on surfaces. A study showed that human influenza viruses retained their infectivity for 24 to 48 h on smooth surfaces (stainless steel, plastic) but for less than 8 to 12 h in porous surfaces such as paper handkerchiefs or news prints [8]. A more recent study describing the survival of two avian respiratory viruses, *i.e.* avian metapneumovirus and avian influenza virus on 12 different surfaces also showed that both survived longer on nonporous surfaces than on porous ones [9]. It was also shown that influenza virus deposited on human hand skin did not survive more than 5 min [8]. A work published in 2007 showed that influenza A/PuertoRico/8/34 (H1N1) virus remained infectious for more than 24 h after spotting on stainless steel surfaces but no more than 6 h on copper [10]. However, H5N1 virus could survive beyond 13 days on glass and steel at 4°C and low relative humidity [11]. Experimental contamination of paper currency with seasonal influenza A(H1N1) and A(H3N2) viruses showed that they can retain their infectivity for several days, especially when viruses were protected by respiratory mucus [12]. Moreover, albeit virus survival was in the same order of magnitude, variations exist between virus isolates, including viruses within the same subtype [6], [13]. Thus, as results obtained with A(H1N1) influenza viruses were mainly obtained with vaccine strains, they cannot be completely extrapolated to the H1N1 pandemic (H1N1pdm) virus that emerged in Mexico during the 2009 outbreak before a careful consideration.

The present study was performed to investigate the survival of H1N1pdm on surfaces and in water. The influenza A/Paris/2590/2009 (H1N1)v strain was subjected to various environmental parameters over time and tested for infectivity using a microtitre endpoint titration. Genomic RNA concentration was also determined by RT-PCR and the integrity of the viral genome was evaluated by a RT-PCR on the whole M segment. The seasonal influenza A/New Caledonia/20/99 (H1N1) virus was also studied in parallel in order to compare the results with those obtained with H1N1pdm strain. The 3 temperatures tested, 4°C, 25°C and 35°C, represented cold, temperate and hot climates respectively. Influenza virus survival can also be influenced by other environmental conditions such as the presence of salt in the medium [14]. In order to evaluate the effect of salinity on H1N1pdm persistence, viruses were diluted in distilled water adjusted with sodium chloride. Four initial concentrations of salt were evaluated: 0 parts per thousand (ppt) (salinity of freshwaters), 5 ppt (salinity of the northern part of the Baltic Sea), 35 ppt (average salinity of oceans) and 270 ppt (salinity of the Dead Sea and salinity in the order of those used in some food processing such as salted pork).

## Materials and Methods

### Cells and viruses

Madin Darby canine kidney (MDCK) cells are a continuous cell line established from kidney of normal female adult Cocker Spaniel in 1958 by SH Madin and NB Darby. They have been kindly given by the French National Influenza Centre for Northern France through the WHO influenza collaborative network. MDCK cells were maintained in Minimum Essential Medium (MEM 1X, GIBCO, Invitrogen), supplemented with 10% fetal calf serum (FCS), antibiotics (0.1 units penicillin, 0.1 µg streptomycin/mL, GIBCO, Invitrogen) and tricine (10 mM, Sigma) at 37°C in humidified 5% CO_2_ incubator.

The seasonal influenza A/New Caledonia/20/99 (H1N1) strain was grown on embryonated chicken eggs and, after reception in our laboratory, twice or 3 times on MDCK cells for water or surface experiments respectively. The pandemic A/Paris/2590/2009 (H1N1)v strain was propagated twice on MDCK cells. Both viruses were grown on MDCK cells without FCS at 35°C for 3 days. The clarified supernatants were harvested, aliquoted and stored at -80°C. The viral titres were determined by endpoint titration as described below. These titres were 10^7.50^ or 10^6.67^ Tissue Culture Infectious Dose 50 per mL (TCID_50_/mL), corresponding to two harvests of the same virus, for the A/New Caledonia/20/99 (H1N1) strain and 10^8.28^ TCID_50_/mL for the A/Paris/2590/2009 (H1N1)v strain.

### Infectivity assays

Endpoint titration was carried out on MDCK cells grown in 96-well plates. Monolayers of MDCK cells in 75 cm^2^ tissue culture flasks were trypsinized with trypsin-EDTA solution (1X, GIBCO, Invitrogen). A cell suspension with a concentration of 3.5×10^5^ cells per milliliter was prepared and 100 µL were seeded on each well of microtitre plates. After 24–36 h, subconfluent monolayers of MDCK cells were washed once with Dulbecco's phosphate buffered saline (DPBS, GIBCO, Invitrogen), and FCS-free medium was added and left until infection.

Viral suspensions were diluted from 10^−1^ to 10^−10^ in FCS-free MEM in round bottom 96-well plates. One hundred microliters of each viral dilution were transferred to 4 rows of 96-well plates containing subconfluent MDCK monolayers, and plates were left at room temperature during 45 min. Then, 100 µL of FCS-free MEM containing trypsin TPCK at a final concentration of 1 µg/mL (Trypsin TPCK treated, Whortington Biochemical Corporation) were added. Plates were covered and incubated at 35°C under 5% CO_2_. After 5 to 7 days, examination for cytopathic effect was performed with light microscopy. TCID_50_ values were calculated according to Reed and Muench's method [15]. The minimal detectable limit of these assays was 10^1.67^ TCID_50_/mL. For assays using saline solution at 270 ppt, salt cytotoxicity increased the limit of virus detection from 10^1.67^ to 10^2.67^ TCID_50_/mL.

### Experimental procedure for trials in water

Distilled water for injectable preparation at a measured pH of 6.5 was used for experiments dealing with the survival of H1N1 viruses in water. The effect of four initial levels of salinity (0, 5, 35 and 270 ppt) on viral survival was evaluated at two temperatures (4 and 35°C). Salinity was adjusted using sodium chloride (NaCl, Fluka, Sigma). Saline concentrations of 0, 5, 35 and 270 ppt were selected to represent natural saline environments. Each virus preparation was diluted 1:10 in saline water samples. The inoculated water samples were then divided into 0.5 mL aliquots in 2 mL polystyrene tubes and placed in an incubator at 35°C or in a refrigerator at 4°C. For each experiment, 2 aliquots were made for parallel titration. At all time points, titration was performed using 4 rows per sample. After titration, samples were stored at −80°C for use in RT-PCR assays. Titre values obtained after the virus suspension was diluted and left under the different conditions during a short period of 30 min, which was arbitrarily chosen, were named “T_0_”. “T_theoretical_” corresponded to the tenth of the titre of the viral stock.

### Experimental procedure for trials on surfaces

Watch glasses (Duran, Fisher Scientific) were used to mimic smooth and chemically inert surfaces which have been shown to avoid loss of virus during recovery (unpublished data). Fifty microliters of initial viral suspension were put on dry-cleaned sterile watch glasses, which were placed into a sealed box (GENbox Jar, bioMerieux) containing silica gel with colorimetric moisture indicator (Chamaleon, VWR BDH Prolabo). Three temperatures were tested: 4, 25 and 35°C. Recovery was performed in 3 steps in the following sequence: i/ by adding 450 or 500 µL of FCS-free medium depending on how dry the surface was, ii/ by scraping the glass surface three times vertically and horizontally using a plastic bacteria inoculation loop, iii/ by mixing three times with a pipette. For each condition, two assays on watch glasses were made for parallel titration and titres were estimated by TCID_50_ method, as described above. Titres obtained after viral suspension was inoculated onto the surface and left 30 min under different temperatures were called “T_wet_”. Titres obtained when the viral suspension was completely dry were called “T_0_”. For each temperature, relative humidity (RH) value was measured once at the beginning of the experiment with a hygrometer/thermometer (Fisherbrand, Fisher Scientific) placed in the sealed box.

### RNA extraction and amplification

Viral RNA was extracted using either a Nucleospin 96 virus kit (Macherey-Nagel) or a Nucleospin RNA Virus kit (Macherey-Nagel) according to the manufacturer's instructions for quantitative real-time RT-PCR (qRT-PCR) or for two steps RT-PCR respectively. Quantitative RT-PCR targeting the M gene was carried out using primers and probes developed by the French National Influenza Reference Centers with a LightCycler 480 instrument (Roche) and a SuperScript III Platinum OneStep RT-PCR kit (Invitrogen) (http://www.who.int/influenza/resources/documents/molecular_diagnosis_influenza_virus_humans_update_201108.pdf). Viral genome integrity was evaluated by two steps endpoint RT-PCR targeting the M gene using a MasterCycler (Eppendorf) thermocycler. Reverse transcription was carried out using SuperScript III First-Strand kit (Invitrogen) and primer annealing in the untranslated genomic region (UTR). PCR was performed with MPBio Taq CORE kit (MP Bio). Sequences of the primers and probes used in these experiments are summarised in Table 1.

**Table 1:** Sequences of the primers and probe used in the qRT-PCR and the RT-PCR.

	A/NewCaledonia/20/99 (H1N1)	A/Paris/2590/2009(H1N1)v
qRT-PCR	**F**: CTTCTAACCGAGGTCGAAACGTA	
	**R**: GGTGACAGGATTGGTCTTGTCTTTA	
	**Probe**: TCAGGCCCCCTCAAAGCCGAG	
RT-PCR	**RT**: AGCAAAAGCAGG	
	**F**: AGCAAAAGCAGGTAGATATTG	**F**: AGCAAAAGCAGGTAGATATTT
	**R**: AGTAGAAACAAGGTAGTTTTT	**R**: AGTAGCAACAAGGTAGTTTTT

RT: primer used for reverse transcription.

F: forward primer.

R: reverse primer.

### Measurement of viral persistence

The freeware Add-in for Microsoft Excel GInaFiT (Geeraerd and Van Impe Inactivation Model Fitting Tool) was used to determine the most suitable microbial survival model to our experimental data [16]. By comparison to the Mean Sum of Squared Errors (MSE), a log-linear model was more suitable. The MSE corresponded to the sum of the squared differences between the experimental data and the identified model (log 10-scale) divided by the number of degrees of freedom. Slope (a) and y-intercept (b) of the linear regression straight line (y = ax + b) and the x-intercept values calculated are represented in Table 2 and 3.

**Table 2:** Persistence times estimated using linear regression model for trials in water.

Strain		Salinity level (ppt)	R^2^	Slope (a)	y-intercept (b)	x-intercept (days)	Virucidy (days)
A/Paris/2590/2009 (H1N1)v	4°C	0	0.9358	−0.0056	6.13	1097 [938; 1328]	716
		5	0.9403	−0.0089	6.22	698 [579; 882]	449
		35	0.9818	−0.0378	7.37	195 [173; 225]	106
		270	0.9373	−0.1190	5.64	47 [33; 93]	34
	35°C	0	0.9462	−0.4716	6.61	14 [11]; [21]	8
		5	0.9927	−0.4101	6.61	16 [14]; [20]	10
		35	0.9964	−0.6825	6.70	10 [9]; [11]	6
A/NewCaledonia/20/99 (H1N1)	4°C	0	0.9627	−0.0066	6.68	1010 [876; 1200]	605
		5	0.8449	−0.0166	5.79	348 [266; 513]	240
		35	0.8565	−0.1412	5.90	42 [32; 63]	28
		270	0.6609	−0.0991	5.16	52 [33; 156]	40
	35°C	0	0.9593	−0.4536	5.84	13 [11]; [16]	9
		5	0.9623	−0.4409	6.15	14 [12]; [17]	9
		35	0.9292	−1.4375	6.27	4 [3]; [7]	3

Slope (a), y-intercept (b) and x-intercept values of the linear regression straight lines calculated from data generated in water. Virucidy corresponded to the duration necessary to obtain a 4 fold reduction of the titre in log_10_.

**Table 3:** Persistence times estimated using linear regression model for trials on surfaces.

Strain		R^2^	Slope(a)	y-intercept (b)	x-intercept (days)	Virucidy(days)
A/Paris/2590/2009 (H1N1)v	4°C	0.8617	−0.09	6.27	66 [33; NA]	42
	25°C	0.4995	−0.19	4.26	22 [13; 145]	21
	35°C	0.9822	−0.43	2.98	7 [5]; [11]	9
A/NewCaledonia/20/99 (H1N1)	4°C	0.0009	−0.01	5.35	2096 [38; NA]	1568
	25°C	0.8033	−0.23	4.30	19 [12; 91]	18
	35°C	0.6523	−0.32	3.35	9 [4; NA]	12

NA: not available.

Slope (a), y-intercept (b) and x-intercept values of the linear regression straight lines calculated from data generated on glass surface (B). Virucidy corresponded to the duration necessary to obtain a 4 fold reduction of the titre in log_10_.

## Results

### Effect of initial change on virus infectivity

**Figure 1:**
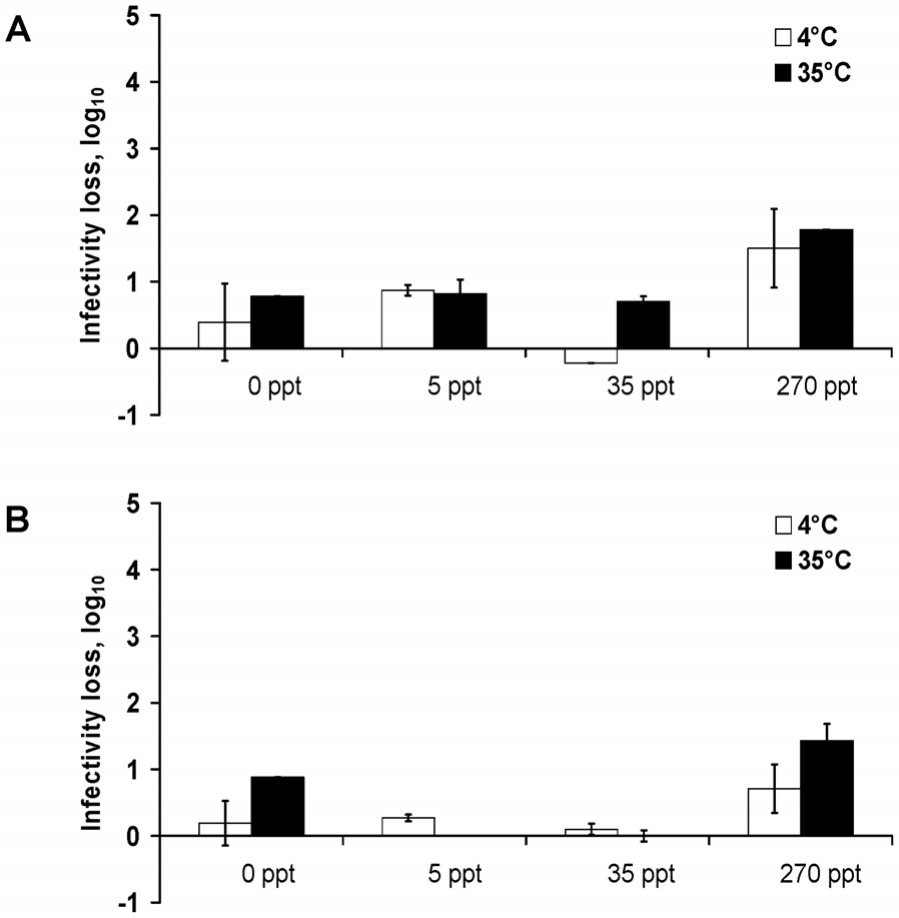
Effect of the initial change on virus infectivity in water. Loss of infectivity during the first 30 min in water for A/Paris/2590/2009 (H1N1)v (A) and A/New Caledonia/20/99 (H1N1) (B). The infectivity loss represented the difference between the T_theoretical,_ *i.e.* 10^7.28^ TCID_50_/mL, and the mean titre obtained after 30 min (T_0_), according to different conditions of temperature and salinity. The mean titre corresponded to the titres made in parallel from the 2 aliquots of water samples. Maximal and minimal values are represented as error bars.

Viral persistence of H1N1pdm and seasonal H1N1 strains was studied once the virus was confined to liquid medium (*i.e.* water in our study) or air (*i.e.* after completely drying of the virus on watch glass). The persistence of A/Paris/2590/2009 (H1N1)v in water was evaluated at two temperatures: 4 and 35°C and with four different salinity conditions. For each condition, viral suspension with a titre of 10^8.28^ TCID_50_/mL was first diluted 1:10 in distilled water with the appropriate amount of salt and then left for 30 min at the studied temperatures before performing endpoint titrations (titre called “T_0_”). The infectivity loss generated during those 30 min was estimated by calculating the difference between the tenth of the titre of the viral stock (called “T_theoretical_” which corresponded to 10^7.28^ TCID_50_/mL) and the T_0_. The loss of infectivity after 30 min was between 0.4 and 0.9 log_10_ for salinity levels of 0, 5 and 35 ppt, but was higher for a salinity of 270 ppt, being of 1.5 and 1.8 log_10_ at 4°C and 35°C respectively (Figure 1A). Similar results were obtained for the A/New Caledonia/20/99 strain (Figure 1B).

On watch glass, infectivity loss was evaluated: i) before the virus droplet spotted on the surface was dry, after 30 min. It corresponded to the difference between the T_theoretical_ and the titre obtained after 30 min, called “T_wet_” ii) it was also evaluated after the virus droplet was completely dry. It corresponded to the difference between the T_theoretical_ and the titre obtained when the viral suspension was totally dry, called “T_0_”. Drying time varied with temperature (17 h40, 7 h45 and 5 h15 at 4, 25 and 35°C respectively).

**Figure 2:**
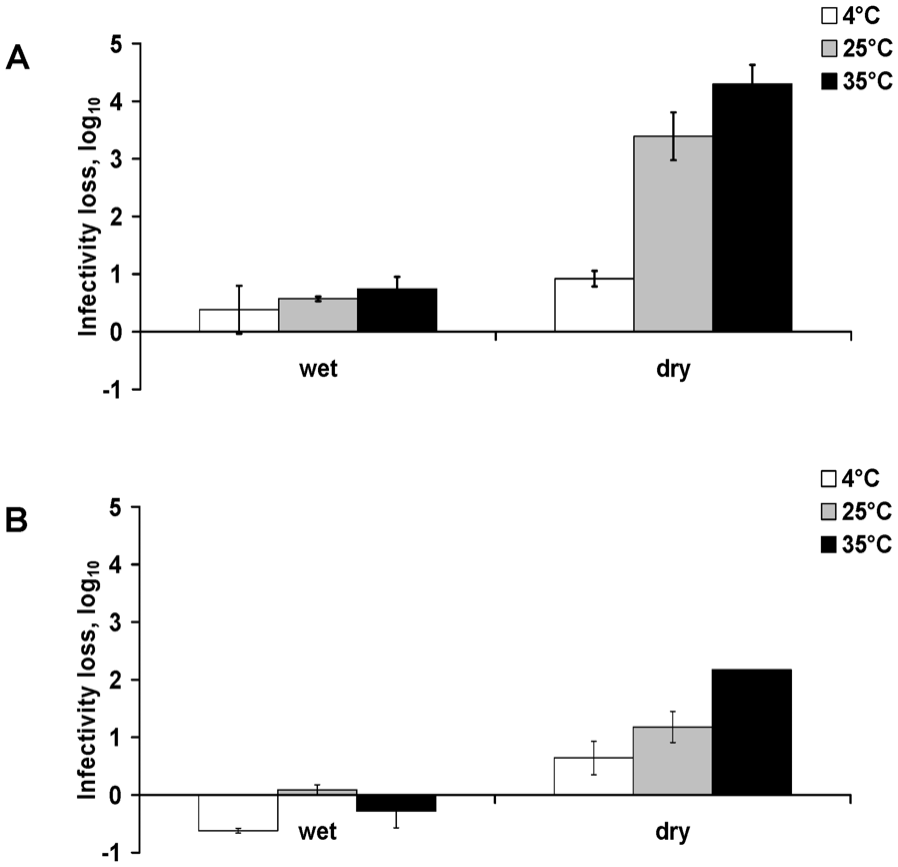
Effect of the initial change on virus infectivity on watch glass. Loss of infectivity during the first 30 min (wet) and after complete drying of the viral suspension (dry) on watch glass for A/Paris/2590/2009 (H1N1)v (A) and A/New Caledonia/20/99 (H1N1) (B) strains. The infectivity loss represented i) the difference between the T_theoretical_ and the mean titre observed after 30 min (T_wet_), and ii) the difference between the T_theoretical_ and the mean titre obtained after complete drying of the viral suspension (T_0_). The mean titre corresponded to the titres made in parallel from the 2 watch glass samples. Maximal and minimal values are represented as error bars.

After 30 min on watch glass, the loss of infectivity compared to T_theoretical_ for H1N1pdm was 0.38, 0.57 and 0.74 log_10_ at 4, 25 and 35°C respectively, corresponding to the range of values obtained in water (Figure 2A). However, after the virus droplet was dry, the loss of infectivity was 3.39 and 4.3 log_10_ at 25°C and 35°C respectively. Similar results were obtained with the seasonal H1N1 virus except for loss of infectivity during drying of the droplet, which was less important than for the H1N1pdm virus (1.18 and 2.17 log_10_ at 25°C and 35°C respectively) (Figure 2B).

### Virus survival in water

**Figure 3:**
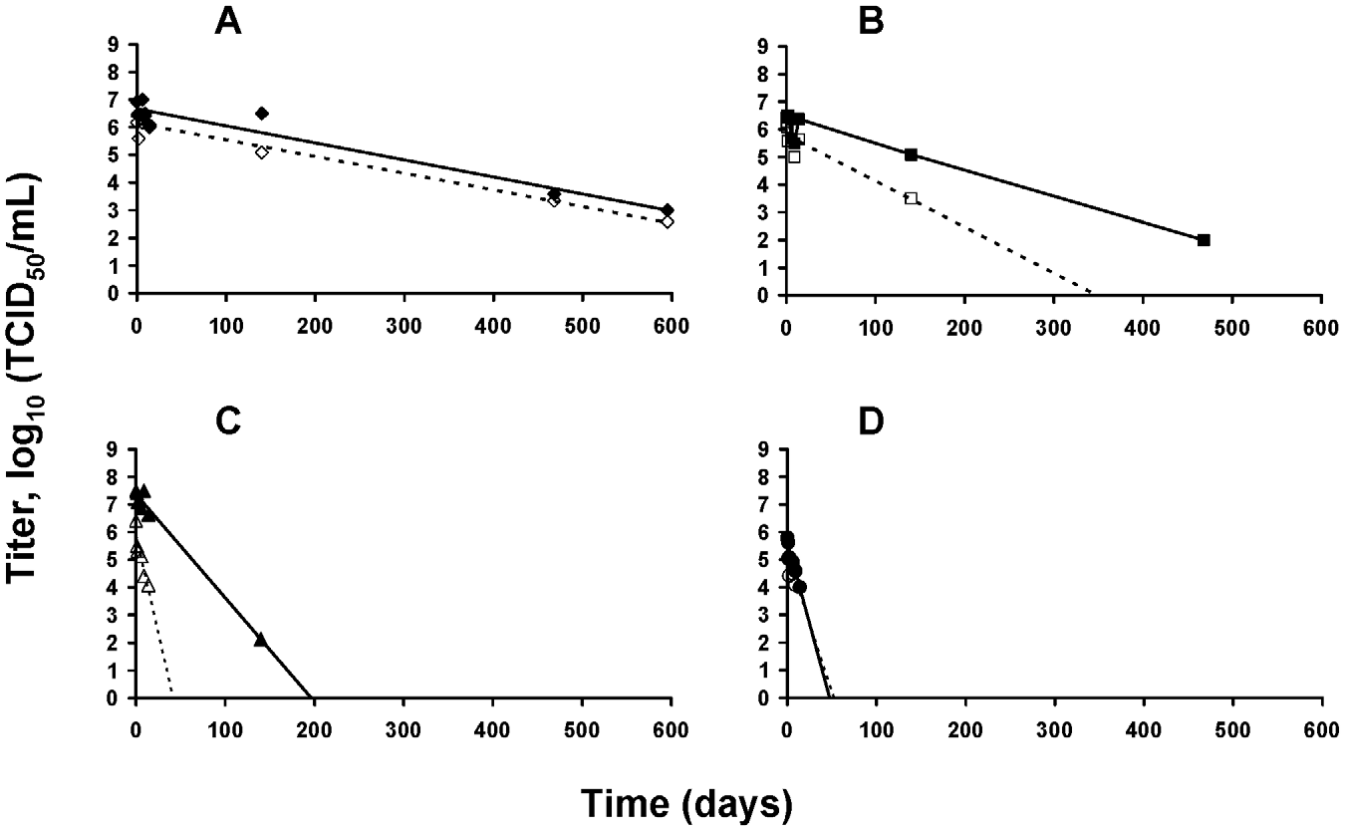
Viral survival in water at 4°C. Viral persistence of A/Paris/2590/2009 (H1N1)v (—) and A/New Caledonia/20/99 (H1N1)(····) in water at 4°C. Linear regression for persistence at 0 ppt (A), 5 ppt (B), 35 ppt (C) and 270 ppt (D) are represented. TCID_50_ values corresponded to the mean values of the titres made in parallel from the 2 aliquots of water samples.

**Figure 4:**
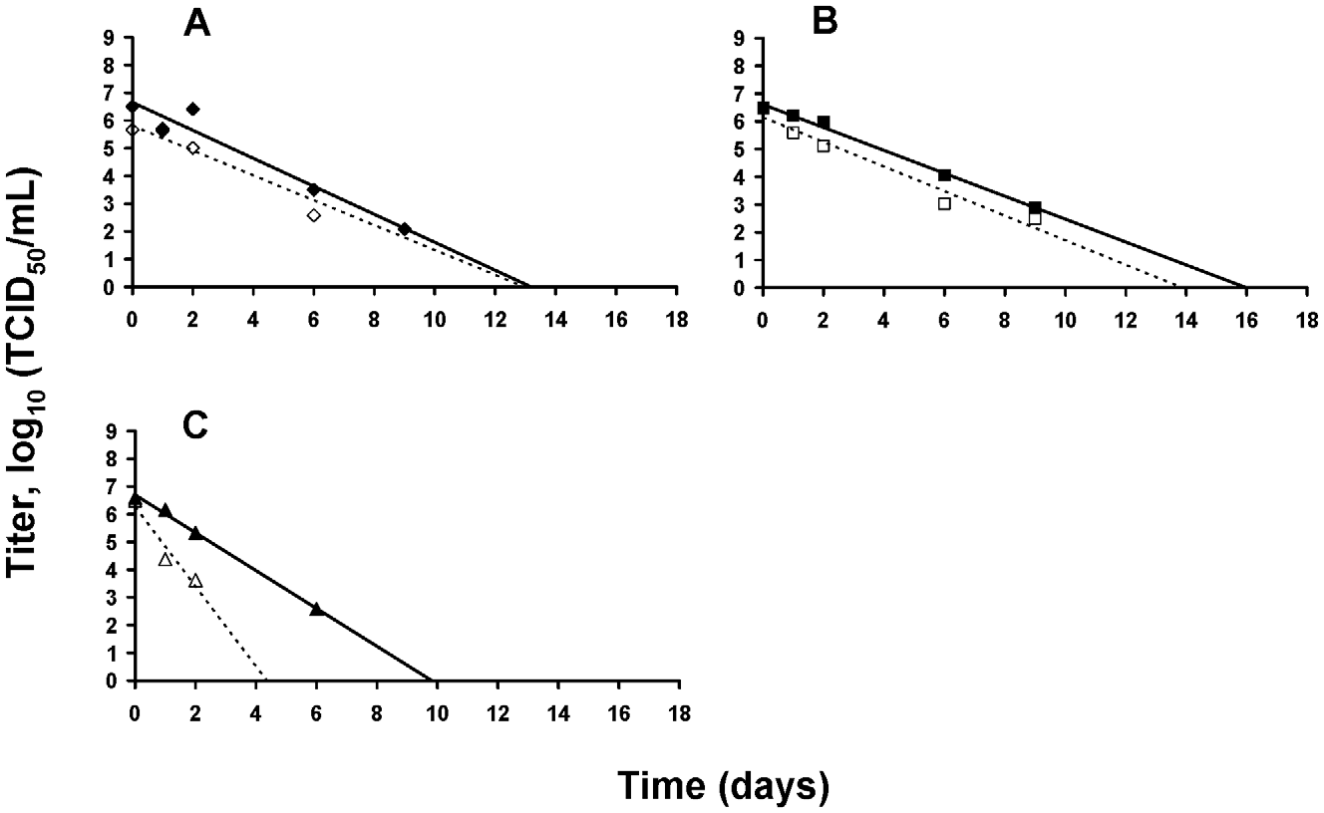
Viral survival in water at 35°C. Viral persistence of A/Paris/2590/2009(H1N1)v (—) and A/New Caledonia/20/99 (H1N1) (····) in water at 35°C. Linear regression for persistence of H1N1pdm in water at 0 ppt (A), 5 ppt (B), and 35 ppt (C) are represented. TCID_50_ values corresponded to the mean values of the titres made in parallel from the 2 aliquots of water samples.

The persistence of A/Paris/2590/2009 (H1N1)v and A/New Caledonia/20/99 (H1N1) in water was evaluated at two temperatures: 4 and 35°C and with four different salinity conditions. Viral suspension diluted 1:10 in distilled water with initial concentrations of 0, 5, 35 or 270 ppt of salt was left for 595 days at 4°C and 14 days at 35°C. Aliquots were periodically removed, to perform endpoint titrations as described in the materials and methods section. TCID_50_/mL values are all available in Table S1. For each condition, kinetics represented in figure 3 and 4 started from T_0_ and TCID_50_ values corresponded to the mean values of the titres made in parallel from the 2 aliquots of water samples. Linear regressions calculated from the experimental values obtained at 4°C and 35°C are also represented in figures 3 and 4 respectively. The linear regression was then used to estimate the x-intercept value which corresponded to the duration beyond which there was no more infectious virus (Table 2).

At low temperature, the H1N1pdm virus was very stable even with a high level of salinity. During the first 14 days, the loss of infectivity was lower than 1 log_10_ for salinity levels of 0, 5 and 35 ppt (Figure 3A, B and C respectively) and around 2 log_10_ for a salinity of 270 ppt (Figure 3D). Almost 5 months later, virus still persisted in water at low salinities (0 and 5 ppt) but lost in part or totally its viability when exposed to higher levels of salinity. In water, at 4°C, based on the modeled values, the H1N1pdm virus would retain its infectivity at 270 ppt up to the extrapolated value of 47 days (Table 2). For lower levels of salinity, this duration was estimated between 195 (for 35 ppt of salt) and 1097 days (for 0 ppt of salt). Differences in persistence estimates were observed between the two strains at low temperature for salinities of 5 and 35 ppt. At 4°C, based on model projections, seasonal H1N1 virus could potentially retain its infectivity up to 348 days instead of 698 for H1N1pdm at 5 ppt and up to 42 days instead of 195 days at 35 ppt, suggesting that H1N1pdm virus is more stable in water than seasonal H1N1 virus. Increasing environmental temperature to 35°C had a negative effect on the survival of the A/Paris/2590/2009 (H1N1)v strain. During the first two days, the loss of infectivity ranged from 0.09 to 1.26 log_10_ for the lowest levels of salinity (0, 5 and 35 ppt). At the sixth day, it ranged from 2.38 to 4 log_10_. The limit of detection (10^1.67^ TCID_50_/mL) was reached between the seventh and the thirteenth day (Figure 4). At 270 ppt of salt, the viral titre decreased by 3 log_10_ and dropped below the detection limit (10^2.67^ TCID_50_/mL; see materials and methods) of the assay in one day only (Table S1). As at 4°C, the x-intercept value was estimated using a log linear regression model. Persistence estimates for H1N1pdm virus ranged between the tenth and the sixteenth day when the level of salinity was of 35, 5 and 0 ppt. At 270 ppt, the time required for a total loss of infectivity was reached in about one day (Tables 2 and S1). At 35°C and 35 ppt of salt, H1N1pdm persisted 10 days in water against 4 days for seasonal H1N1 virus (Table 2), reinforcing the idea that H1N1pdm virus is more stable than seasonal H1N1 virus. Because initial T_0_ were different for both virus strains, we determined virucidy from the slopes calculated from linear regression (Table 2).

**Figure 5:**
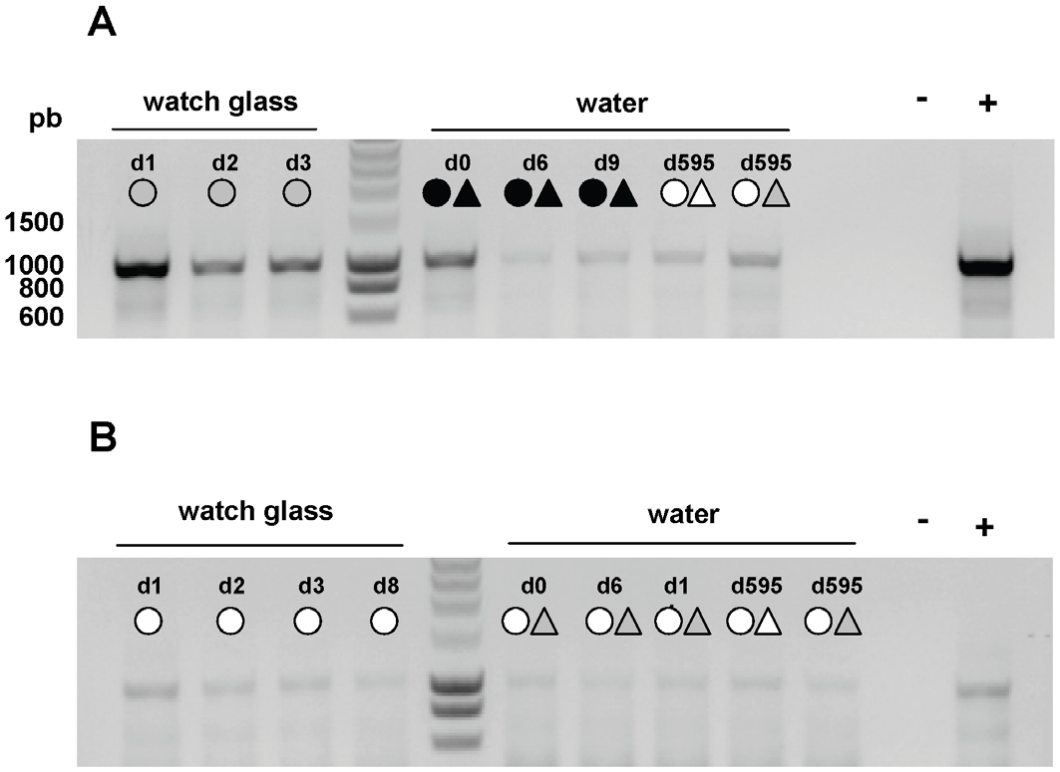
Endpoint RT-PCR targeting the whole M segment. RT-PCR was performed for A/Paris/2590/2009 (H1N1)v (A) and A/New Caledonia/20/99 (H1N1) (B) and for selected conditions: at different days (designated as dx, x being the number of the day), different temperatures (for 4, 25 and 35°C respectively) and different levels of salinity (for 0, 35 and 270 ppt respectively). **+**: initial stock of A/Paris/2590/2009 (H1N1)v or A/New Caledonia/20/99 (H1N1) strain as a positive control. **–**: water as a negative control.

The pH of water containing diluted virus was checked over time and found to be very stable at 6.9 for each condition. From each aliquot sample used to estimate TCID_50_/mL values, viral RNA was also extracted in order to quantify genome copy numbers. Real-time RT-PCR targeting the M gene was performed as described in the Materials and Methods section. Results giving the genome copy numbers for each experimental condition are shown in Table 4. In water, at 4°C and 35°C, genomic RNA concentration obtained over time was compared to that obtained at the beginning of the kinetic, i.e. after 30 min in water. Relative values varied from 95% to 105%, showing that RNA genome quantity remained stable. The RT-PCR targeting the whole M segment was also performed for few conditions and the results presented in Figure 5 showed that a large part of RNA genome remained intact in virus particles.

**Table 4:** M genomic segment concentrations for trials in water.

Strain		Salinitylevel (ppt)	d_theoretical_	d0	d1	d2	d6	d9	d14	d140	d595
A/Paris/2590/2009 (H1N1)v	4°C	0	9.74	10.15	10.26	NA	10.71	NA	9.79	9.83	9.30
				10.13	10.19	NA	10.68	NA	9.85	9.60	NA
		5	9.74	10.02	10.29	NA	10.62	NA	9.92	9.01	NA
				10.13	10.18	NA	10.58	NA	9.81	9.56	NA
		35	9.74	10.82	10.95	NA	11.01	NA	10.17	9.73	9.65
				10.78	10.79	NA	10.97	NA	10.14	10.03	NA
		270	9.74	10.48	10.64	NA	11.00	NA	10.33	10.28	NA
				10.49	10.53	NA	10.89	NA	10.15	10.29	NA
	35°C	0	9.74	10.00	10.06	10.26	9.62	9.89	NA	NA	NA
				10.12	9.94	9.37	9.65	9.58	NA	NA	NA
		5	9.74	10.04	8.97	10.50	10.33	9.76	NA	NA	NA
				10.08	9.48	10.36	8.97	9.60	NA	NA	NA
		35	9.74	10.57	10.47	10.42	10.38	10.21	NA	NA	NA
				10.30	10.21	10.46	10.18	9.98	NA	NA	NA
		270	9.74	10.39	10.41	10.57	10.56	10.80	NA	NA	NA
				10.36	10.44	10.60	10.60	10.66	NA	NA	NA
A/NewCaledonia/20/99 (H1N1)	4°C	0	NA	9.94	10.04	NA	10.15	NA	9.40	8.58	NA
				9.60	10.05	NA	10.27	NA	9.96	8.70	8.15
		5	NA	8.92	9.23	NA	9.40	NA	8.95	8.35	NA
				9.10	9.19	NA	9.63	NA	8.98	8.21	NA
		35	NA	10.15	10.35	NA	10.66	NA	10.33	9.38	NA
				10.18	10.48	NA	10.71	NA	10.41	9.15	8.60
		270	NA	9.87	10.06	NA	10.94	NA	10.23	10.18	NA
				10.01	10.15	NA	10.78	NA	10.32	10.18	NA
	35°C	0	NA	10.10	9.31	9.52	8.72	8.93	7.96	NA	NA
				9.49	9.24	9.49	8.76	8.82	8.19	NA	NA
		5	NA	8.13	9.02	9.53	8.98	8.42	7.42	NA	NA
				9.04	8.91	9.32	8.76	8.96	7.03	NA	NA
		35	NA	10.11	9.55	9.62	8.85	8.96	7.36	NA	NA
				10.13	9.51	9.83	9.07	8.92	8.16	NA	NA
		270	NA	9.40	9.80	10.90	10.81	10.76	10.18	NA	NA
				9.90	10.35	10.96	10.94	10.71	9.95	NA	NA

NA: not available.

M genomic segment concentrations expressed in log(copy number/mL) obtained for A/Paris/2590/2009 (H1N1)v and A/New Caledonia/20/99 (H1N1) strains in water. Concentrations were determined at different days (designated as dx, x being the number of the day). RNA concentration at d_theoretical_ was obtained after the viral stock was diluted 1:10. In water (A), RNA concentration at d_0_ was obtained after the viral suspension was left 30 min in water under different conditions. On watch glass (B), RNA concentration at d_wet_ was obtained 30 min after the viral suspension was left on the surface and RNA concentration at d_0_ was obtained after this viral suspension was totally dry. All experiments were done in duplicate.

### Viral survival on smooth surface

**Figure 6:**
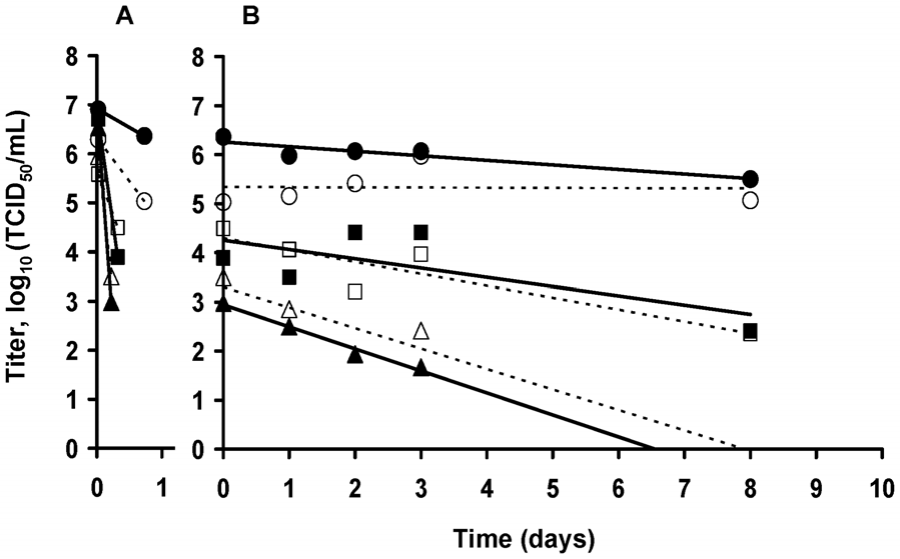
Viral survival kinetic on watch glass. Viral persistence of A/Paris/2590/2009 (H1N1)v (—) and A/New Caledonia/20/99 (H1N1) (····) at 4 (•;○), 25 (▪;□) and 35°C (▴;Δ). Linear regressions for persistence during drying (A) and after drying (B) are represented. TCID_50_ values corresponded to the mean values of the titres made in parallel from the 2 watch glass samples.

The persistence of both H1N1 viral strains on surface was tested on watch glasses and evaluated at three temperatures (4, 25 and 35°C). The TCID_50_ titre evaluated as soon as the virus spotted on the surface was dry was labeled “T_0_” and represented the starting point of the kinetics of persistence of viable virus on watch glass. Initial relative humidity values measured at 4, 25 and 35°C were 28, 16 and 12% respectively. In 8 days, at 4°C and 25°C, the titre decreased by 1 log_10_ and 1.5 log_10_ respectively. At 35°C, a loss of 1.5 log_10_ was also observed but in 3 days. TCID_50_/mL values are all available in Table S1. Losses of infectivity during the drying and linear regressions calculated after it are represented in Figure 6A and 6B respectively. In order to estimate the persistence in a dry droplet of the H1N1pdm spotted on a smooth surface, x-intercept values were determined as described above for each temperature. Modeled data suggested that after 66, 22 and 7 days no more infectious particle would be present at 4, 25 and 35°C respectively (Table 3). Similar results were obtained for A/New Caledonia/20/99 (H1N1) virus strain, except at 4°C. The R^2^ obtained for the regression linear model was very low as it was calculated on a set of variable values measured in a number insufficient for such a slow loss of infectivity. This very low R^2^ illustrated the unreliability of the extrapolated value of 2096 days.

As observed in water studies, genomic RNA relative concentration was also very stable and varied between 98 and 100% (Table 5). RT-PCR targeting the whole M segment showed again that a large part of the RNA genome remained intact in virus particles, even after the deleterious drying step of the virus (Figure 5).

**Table 5:** M genomic segment concentrations for trials on surfaces.

Strain		d_theoretical_	d_wet_	d0	d1	d2	d3	d8
A/Paris/2590/2009 (H1N1)v	4°C	9.74	10.19	10.11	10.07	10.01	10.12	10.10
			10.15	10.09	10.14	9.99	10.09	10.14
	25°C	9.74	11.19	10.74	10.75	10.61	10.30	10.74
			10.77	10.58	10.41	10.42	10.57	10.56
	35°C	9.74	10.87	10.41	10.34	10.34	10.16	NA
			10.21	10.35	10.53	10.36	10.39	NA
A/NewCaledonia/20/99 (H1N1)	4°C	NA	10.93	10.22	10.21	10.13	10.09	10.18
			10.69	10.22	10.05	10.07	9.97	10.22
	25°C	NA	10.31	9.83	9.79	9.76	9.68	9.65
			10.37	9.63	9.32	9.29	9.52	9.56
	35°C	NA	9.92	9.52	9.00	9.29	9.28	9.44
			10.68	9.46	9.41	9.38	9.51	9.37

NA: not available.

M genomic segment concentrations expressed in log(copy number/mL) obtained for A/Paris/2590/2009 (H1N1)v and A/New Caledonia/20/99 (H1N1) strains on watch glass (B). Concentrations were determined at different days (designated as dx, x being the number of the day). RNA concentration at d_theoretical_ was obtained after the viral stock was diluted 1:10. In water (A), RNA concentration at d_0_ was obtained after the viral suspension was left 30 min in water under different conditions. On watch glass (B), RNA concentration at d_wet_ was obtained 30 min after the viral suspension was left on the surface and RNA concentration at d_0_ was obtained after this viral suspension was totally dry. All experiments were done in duplicate.

## Discussion

The duration of viral infectivity in water was related to the environmental conditions. Our results showed that the 2009 pandemic A(H1N1) virus had the ability to persist in water and on glass surface for several weeks, even at 35°C. As described previously, low temperature increased virus survival [5]–[7], [17]. At low salinity levels (0 and 5 ppt), maximum estimated survival times varied between 698 and 1097 days. Increasing environmental temperature and salinity level had a strong negative effect on the survival of the H1N1pdm virus which retained its infectivity no more than 1 day at 35°C and 270 ppt of salt. However, for lower levels of salinity, mostly encountered in the environment, the virus could remain infectious for at least 10 days in water. Our persistence estimates are in agreement with previously published data generated with other influenza virus subtypes [13], [18], [19]. Contrary to most results of these previous studies which were extrapolated, our results obtained at 595 days for 4°C were determined by microtitre endpoint titrations. This is consistent with recent work showing that H6N2 virus inactivation rate yield persistence times ranging from 40 days at 37°C to more than 600 days at 4°C [20]. A work based on the study of seven H2N3 isolates showed that virus survival followed a nonlinear decrease in viral titre at 55°C. This two-step model should be observed due to quick succession of experimental points and to this specific temperature [13], which are not the case in our study. Because maximum survival times were dependant on initial viral titres, we also determined the virucidal activity of each parameter, which corresponded to a reduction of 4 log_10_ of the titre according to the European Standards (NF EN 14476) (Table 2 and 3).

Influenza viruses are thought to be transmitted from person to person through direct or indirect contact with respiratory secretions and/or when touching surfaces contaminated with influenza viruses. Persistence of H1N1pdm on the hard non-porous surface of glass showed that this virus survived for prolonged periods of time. Drying of the viral suspension had a strong negative effect on viral infectivity, but after complete drying of the virus inocula, H1N1pdm unexpectedly remained infectious from 1 week at 35°C and up to 2 months at 4°C. Indeed, our results showed that H1N1pdm remained infective for at least 7 days at 35°C and up to 66 days at 4°C, which is compatible with extrapolated data obtained with H5N1 virus [11]. These values are much higher than those previously observed for metal or plastic surfaces [8], [10]. We noted that at 25°C and 35°C, the virucidal activity was almost already reached after drying. However, our data are consistent with the unexpected stability of the human A(H3N2) and A(H1N1) viruses on paper currency [12]. Moreover, virus stability might be even higher in natural settings than in experimental conditions, as addition of mucus was shown to increase virus survival of seasonal H3N2 viruses [12]. As all experimental models, our setting was a simplified version of the real environmental conditions. Distilled water is a sterile environment which does not reflect the possible contamination of water with other biological agents or chemical compounds, such as bacteria or mucus. Indeed, long-lasting persistence times of virus survival described in our study would probably be prolonged by the presence of mucus, as described by Thomas *et al* [12].

As outlined by RT-PCR quantification in different environmental conditions, a constant level of viral RNA was detected over time, suggesting that there was hardly any loss of viral genetic material (Table 4 and 5). As the real-time RT-PCR assay targeted a very small part of viral nucleic acid, genomic RNA integrity was also evaluated by using specific primers targeting a larger region of the M segment (1027 bp). The results showed that the whole genomic RNA segment was detectable on gel electrophoresis suggesting that a significant part of viral RNA was not degraded. Single stranded RNA molecules are rapidly degraded unless they are protected by *ad hoc* structures. In the case of influenza A viruses, native ribonucleoproteins prevent RNases, for example, from damaging viral genome. Therefore, other virus structures must be involved in the loss of viability. M1 not being in direct contact with the environment, external viral structures more directly in contact with the medium are mostly involved in virus loss of infectivity. This has not yet been shown in the literature. One of the explanations is that the HA and NA glycoproteins as well as the M2 protein of both H1N1 viruses could play a role in this process. The structural differences between H1N1pdm virus and seasonal H1N1 virus could be the basis of their different stability in water. The amino-acid composition of the envelope proteins might influence the stability of the overall molecules in given conditions. Moreover, it might also significantly impact the surface electrical potential of the virions with possible consequences in their interaction with chemical and biological compounds of their molecular environment. Between the two N1 proteins, there were 86 amino-acid differences. The HA is the most abundant protein on the virion surface and is pivotal for viral entry. Between the two H1 proteins, there were 99 and 17 amino-acid differences in the HA1 and HA2 subunits respectively. Three of them affected glycosylation sites and one amino-acid implicated in a hydrogen bond between HA1 and HA2. The amino-acid composition also indicates differences in the isoelectric point of the two proteins (6.6 for the A/NewCaledonia/20/99 (H1N1) strain and 7.6 for the A/Paris/2590/2009 (H1N1)v strain), which might have an impact in the local interactions with other molecules, especially with variable concentration of sodium chloride.

Our results showed that once in water or once dry on a smooth non-porous surface, the H1N1pdm virus retained its infectious power for long periods of time, even at 35°C. Our findings of long-term persistence of H1N1pdm virus in water and on non-porous surface showed that contaminated environments can remain a source of virus for efficient transmission over prolonged periods of time. This is important to elaborate and implement control measures based on basic hygiene: hand-washing, cleaning and disinfecting surfaces such as door-knobs, using of disposable handkerchiefs, wearing masks. This is also important for biosecurity applied to pig farms as the H1N1pdm virus is known to be able to infect this animal species [21].

In water, we have shown that H5N1 virus could also remain infectious for 3 years at 4°C (unpublished data). Lakes most probably play a central role in virus transmission between birds and possibly constitute efficient relays for virus transmission from one year to the other one. The low-temperature environment in certain lakes in Asia including Siberia allow avian influenza viruses to survive in water for period of time long enough to remain infectious when the migratory bird return the following year.

## Supporting Information

Table S1 **Log_10_TCID_50_/mL values.** Log_10_TCID_50_/mL values obtained with A/Paris/2590/2009 (H1N1)v and A/New Caledonia/20/99 (H1N1) strains in water (A) and on watch glass (B). Viral titers were obtained at different days (designated as dx, x being the number of the day). Titer obtained at d_theoretical_ corresponded to the tenth of the viral stock titer. In water (A), the titer calculated at d_0_ corresponded to the titer obtained after the virus suspension was diluted and left under different conditions during 30 min. On watch glass (B), the titer at d_wet_ corresponded to the titer obtained after the viral suspension was left 30 min on the surface and the titer obtained at d_0_ corresponded to the titer calculated after this viral suspension was totally dry. All experiments were done in duplicate.
(DOC)

## References

[1] Tellier R (2006). Review of aerosol transmission of influenza A virus.. Emerging Infect Dis.

[2] Tellier R (2009). Aerosol transmission of influenza A virus: a review of new studies.. J R Soc Interface.

[3] Hinshaw VS, Webster RG, Turner B (1979). Water-bone transmission of influenza A viruses?. Intervirology.

[4] Stallknecht DE, Goekjian VH, Wilcox BR, Poulson RL, Brown JD (2010). Avian influenza virus in aquatic habitats: what do we need to learn?. Avian Dis.

[5] Brown JD, Swayne DE, Cooper RJ, Burns RE, Stallknecht DE (2007). Persistence of H5 and H7 avian influenza viruses in water.. Avian Dis.

[6] Stallknecht DE, Shane SM, Kearney MT, Zwank PJ (1990). Persistence of avian influenza viruses in water.. Avian Dis.

[7] Shahid MA, Abubakar M, Hameed S, Hassan S (2009). Avian influenza virus (H5N1); effects of physico-chemical factors on its survival.. Virol J.

[8] Bean B, Moore BM, Sterner B, Peterson LR, Gerding DN (1982). Survival of influenza viruses on environmental surfaces.. J Infect Dis.

[9] Tiwari A, Patnayak DP, Chander Y, Parsad M, Goyal SM (2006). Survival of two avian respiratory viruses on porous and nonporous surfaces.. Avian Dis.

[10] Noyce JO, Michels H, Keevil CW (2007). Inactivation of influenza A virus on copper versus stainless steel surfaces.. Appl Environ Microbiol.

[11] Wood JP, Choi YW, Chappie DJ, Rogers JV, Kaye JZ (2010). Environmental persistence of a highly pathogenic avian influenza (H5N1) virus.. Environ Sci Technol.

[12] Thomas Y, Vogel G, Wunderli W, Suter P, Witschi M (2008). Survival of Influenza Virus on Banknotes. Appl Environ Microbiol..

[13] Negovetich NJ, Webster RG (2010). http://www.ncbi.nlm.nih.gov/pubmed/20610728.

[14] Stallknecht DE, Kearney MT, Shane SM, Zwank PJ (1990). Effects of pH, temperature, and salinity on persistence of avian influenza viruses in water.. Avian Dis.

[15] Reed LJ, Muench H (1938). A simple method of estimating 50 per cent end-points.. Am J Hyg.

[16] Geeraerd AH, Valdramidis VP, Van Impe JF (2005). GInaFiT, a freeware tool to assess non-log-linear microbial survivor curves.. Int J Food Microbiol.

[17] Davidson I, Nagar S, Haddas R, Ben-Shabat M, Golender N (2010). Avian influenza virus H9N2 survival at different temperatures and pHs.. Avian Dis.

[18] De Benedictis P, Beato MS, Capua I (2007). Inactivation of avian influenza viruses by chemical agents and physical conditions: a review.. Zoonoses and public health.

[19] Stallknecht DE, Brown JD (2009). Tenacity of avian influenza viruses.. Rev - Off Int Epizoot.

[20] Graiver DA, Topliff CL, Kelling CL, Bartelt-Hunt SL (2009). Survival of the Avian Influenza Virus (H6N2) After Land Disposal.. Environ Sci Technol.

[21] Zhou H, Wang C, Yang Y, Guo X, Kang C (2011). Pandemic (H1N1) 2009 Virus in Swine Herds, People's Republic of China.. Emerging Infect Dis.

