# Supplementary material for: Persistence of the 2009 Pandemic Influenza A (H1N1) Virus in Water and on Non-Porous Surface
Source: PLoS One. 2011 Nov 23;6(11):e28043. doi: 10.1371/journal.pone.0028043 (PMC3223208; doi:10.1371/journal.pone.0028043)
Supplement: Table S1 — Log10TCID50/mL values. Log10TCID50/mL values obtained with A/Paris/2590/2009 (H1N1)v and A/New Caledonia/20/99 (H1N1) strains in water (A) and on watch glass (B). Viral titers were obtained at different days (designated as dx, x being the number of the day). Titer obtained at dtheoretical corresponded to the tenth of the viral stock titer. In water (A), the titer calculated at d0 corresponded to the titer obtained after the virus suspension was diluted and left under different conditions during 30 min. On watch glass (B), the titer at dwet corresponded to the titer obtained after the viral suspension was left 30 min on the surface and the titer obtained at d0 corresponded to the titer calculated after this viral suspension was totally dry. All experiments were done in duplicate. (DOC) [file pone.0028043.s001.doc]

**Table S1: Log_10_TCID_50_/mL values**

**A**

|  |  | Salinity level  (ppt) | d_theoretical_ | d0 | d1 | d2 | d6 | d9 | d14 | d140 | d468 | d595 |
| --- | --- | --- | --- | --- | --- | --- | --- | --- | --- | --- | --- | --- |
| A/Paris/2590/2009  (H1N1)v | 4°C | 0 | 7.28 | 6.33 | 6.67 | 6.33 | 7.5 | 6.33 | 6.5 | 6.5 | 3.5 | 3.5 |
|  |  |  |  | 7.5 | 6.25 | 6.5 | 6.5 | 6.5 | 5.67 | 6.5 | 3.67 | 2.5 |
|  |  | 5 | 7.28 | 6.5 | 6.5 | 6.5 | 5.67 | 5.5 | 6.25 | 4.67 | <1.67 | <1.67 |
|  |  |  |  | 6.33 | 6.5 | 6.5 | 5.67 | 5.5 | 6.5 | 5.5 | 2 | <1.67 |
|  |  | 35 | 7.28 | 7.5 | 7.33 | 6.67 | 7.5 | 7.5 | 6.5 | 1.75 | <1.67 | NA |
|  |  |  |  | 7.5 | 7.5 | 7.5 | 6.25 | 7.5 | 6.75 | 2.5 | <1.67 | NA |
|  |  | 270 | 7.28 | 6.39 | 5.5 | 4.67 | 5.5 | 4.67 | 4.33 | <2.5 | NA | NA |
|  |  |  |  | 5.22 | 5.75 | 5.5 | 4.33 | 4.5 | 3.67 | <2.5 | NA | NA |
|  | 35°C | 0 | 7.28 | 6.5 | 5.75 | 6.5 | 4.33 | 2.5 | NA | NA | NA | NA |
|  |  |  |  | 6.5 | 5.67 | 6.33 | 2.67 | <1.67 | NA | NA | NA | NA |
|  |  | 5 | 7.28 | 6.25 | 6.17 | 5.5 | 4.5 | 2.5 | NA | NA | NA | NA |
|  |  |  |  | 6.67 | 6.25 | 6.5 | 3.67 | 3.33 | NA | NA | NA | NA |
|  |  | 35 | 7.28 | 6.67 | 6.5 | 5.33 | 2.5 | <1.67 | NA | NA | NA | NA |
|  |  |  |  | 6.5 | 5.83 | 5.33 | 2.67 | <1.67 | NA | NA | NA | NA |
|  |  | 270 | 7.28 | 5.5 | <2.5 | <2.5 | <2.5 | <2.5 | NA | NA | NA | NA |
|  |  |  |  | 5.5 | <2.5 | <2.5 | <2.5 | <2.5 | NA | NA | NA | NA |
| A/NewCaledonia/20/99  (H1N1) | 4°C | 0 | 6.5 | 6.5 | 6.5 | 5.67 | 6.5 | 6.5 | 5.5 | 4.67 | 3.67 | 2.67 |
|  |  |  |  | 5.83 | 6.33 | 5.5 | 5.83 | 6.5 | 6.5 | 5 | 3 | 2.5 |
|  |  | 5 | 6.5 | 6.33 | 6.5 | 5.67 | 5.67 | 5.5 | 5.63 | 3.5 | <1.67 | <1.67 |
|  |  |  |  | 6.23 | 5.67 | 5.5 | 5.5 | 4.5 | 5.67 | 3.5 | <1.67 | <1.67 |
|  |  | 35 | 6.5 | 6.5 | 5.5 | 5.33 | 5.5 | 4.33 | 3.67 | <1.67 | <1.67 | NA |
|  |  |  |  | 6.33 | 5.5 | 5.33 | 4.75 | 4.5 | 4.5 | <1.67 | <1.67 | NA |
|  |  | 270 | 6.5 | 5.39 | 5.33 | 4.5 | 4.5 | 3.67 | 4.5 | <2.5 | NA | NA |
|  |  |  |  | 6.12 | 4.75 | 4.33 | 4.5 | 4.5 | 3.5 | <2.5 | NA | NA |
|  | 35°C | 0 | 6.5 | 3.67 | 5.5 | 5.5 | 2.67 | 1.67 | <1.67 | NA | NA | NA |
|  |  |  |  | 5.67 | 5.77 | 4.53 | 2.5 | 2.5 | <1.67 | NA | NA | NA |
|  |  | 5 | 6.5 | 6.5 | 5.5 | 5.5 | 3.67 | 2.5 | <1.67 | NA | NA | NA |
|  |  |  |  | 6.5 | 5.67 | 4.75 | 2.5 | 2.5 | <1.67 | NA | NA | NA |
|  |  | 35 | 6.5 | 6.5 | 4.25 | 3.75 | <1.67 | <1.67 | <1.67 | NA | NA | NA |
|  |  |  |  | 6.5 | 4.5 | 3.5 | <1.67 | <1.67 | <1.67 | NA | NA | NA |
|  |  | 270 | 6.5 | 5.33 | <2.5 | <2.5 | <2.5 | <2.5 | <2.5 | NA | NA | NA |
|  |  |  |  | 4.83 | <2.5 | <2.5 | <2.5 | <2.5 | <2.5 | NA | NA | NA |

**B**

|  |  | d_theoretical_ | d_wet_ | d0 | d1 | d2 | d3 | d8 |
| --- | --- | --- | --- | --- | --- | --- | --- | --- |
| A/Paris/2590/2009  (H1N1)v | 4°C | 7.28 | 7.33 | 6.23 | 5.5 | 6.5 | 5.67 | 5.5 |
|  |  |  | 6.5 | 6.5 | 6.5 | 5.67 | 6.5 | 5.5 |
|  | 25°C | 7.28 | 6.67 | 3.5 | 3.5 | 4.33 | 4.5 | 2.5 |
|  |  |  | 6.75 | 4.33 | 3.5 | 4.5 | 4.33 | 2.33 |
|  | 35°C | 7.28 | 6.33 | 2.67 | 2.5 | <1.67 | 1.67 | NA |
|  |  |  | 6.75 | 3.33 | 2.5 | 2.25 | 1.67 | NA |
| A/NewCaledonia/20/99  (H1N1) | 4°C | 5.67 | 6.25 | 4.75 | 4.83 | 5.5 | 5.5 | 5.5 |
|  |  |  | 6.33 | 5.33 | 5.5 | 5.33 | 6.5 | 4.67 |
|  | 25°C | 5.67 | 5.67 | 4.23 | 4.5 | 2.75 | 3.5 | 3.33 |
|  |  |  | 5.5 | 4.77 | 3.67 | 3.75 | 4.5 | 1.67 |
|  | 35°C | 5.67 | 5.67 | 3.5 | 3.5 | 2.23 | 1.67 | <1.67 |
|  |  |  | 6.25 | 3.5 | 2.33 | <1.67 | NA | <1.67 |
